# The sensitivity of biological finite element models to the resolution of surface geometry: a case study of crocodilian crania

**DOI:** 10.7717/peerj.988

**Published:** 2015-06-02

**Authors:** Matthew R. McCurry, Alistair R. Evans, Colin R. McHenry

**Affiliations:** 1 Department of Anatomy and Developmental Biology, Monash University, Clayton, Melbourne, Australia; 2 Geosciences, Museum Victoria, Carlton, Melbourne, Australia; 3 School of Biological Sciences, Monash University, Clayton, Melbourne, Australia; 4 School of Engineering, University of Newcastle, Callaghan, Australia

**Keywords:** Finite element analysis, Biomechanics, Resolution, Skull, Sensitivity

## Abstract

The reliability of finite element analysis (FEA) in biomechanical investigations depends upon understanding the influence of model assumptions. In producing finite element models, surface mesh resolution is influenced by the resolution of input geometry, and influences the resolution of the ensuing solid mesh used for numerical analysis. Despite a large number of studies incorporating sensitivity studies of the effects of solid mesh resolution there has not yet been any investigation into the effect of surface mesh resolution upon results in a comparative context. Here we use a dataset of crocodile crania to examine the effects of surface resolution on FEA results in a comparative context. Seven high-resolution surface meshes were each down-sampled to varying degrees while keeping the resulting number of solid elements constant. These models were then subjected to bite and shake load cases using finite element analysis. The results show that incremental decreases in surface resolution can result in fluctuations in strain magnitudes, but that it is possible to obtain stable results using lower resolution surface in a comparative FEA study. As surface mesh resolution links input geometry with the resulting solid mesh, the implication of these results is that low resolution input geometry and solid meshes may provide valid results in a comparative context.

## Introduction

Comparative biomechanics applies mechanical theory and techniques to better understand differences between biological structures and systems (Vogel, 2003). Finite element analysis (FEA) is a computational modelling technique that has become a widely used method in comparative biomechanics. The use of this method in anatomical studies (whether biological, palaeontological or medical) has allowed for a better understanding of how variation in morphology, material properties and loading conditions can influence the strength of a structure (Chen et al., 2012; Degrange et al., 2010; Gröning, Fagan & O’Higgins, 2011; Lautenschlager et al., 2013; McHenry et al., 2006; McHenry et al., 2007; Omasta et al., 2012; Rayfield, 2004; Snively & Theodor, 2011; Tseng, 2013; Walmsley et al., 2013b; Wroe, 2008). FEA uses differential equations to approximate the patterns of stress and strain that would occur in a structure under a specified set of loading and boundary conditions (Cook et al., 2001). It provides a useful means to test the influence of anatomical variation on structural performance and hence understand the functional reasons driving the evolution of certain anatomical traits. Scientific modelling aims to represent physical objects or phenomena in a simplified, logical way. Following this, an ideal model is not necessarily the most complex but instead the simplest model that is useful for a given purpose. By making assumptions the model can be simplified to a greater degree; for example in studies using finite element analysis to examine variation in skeletal structure, the material properties of bone are frequently modelled as a homogeneous material rather than a heterogeneous material. However, it is important that the influence of each assumption is understood in order to determine whether the results are still reliable. Here we aim to use a dataset of crocodile crania to examine an underappreciated factor in FEA model construction: the resolution of the surface mesh prior to solid meshing.

**Figure 1:**
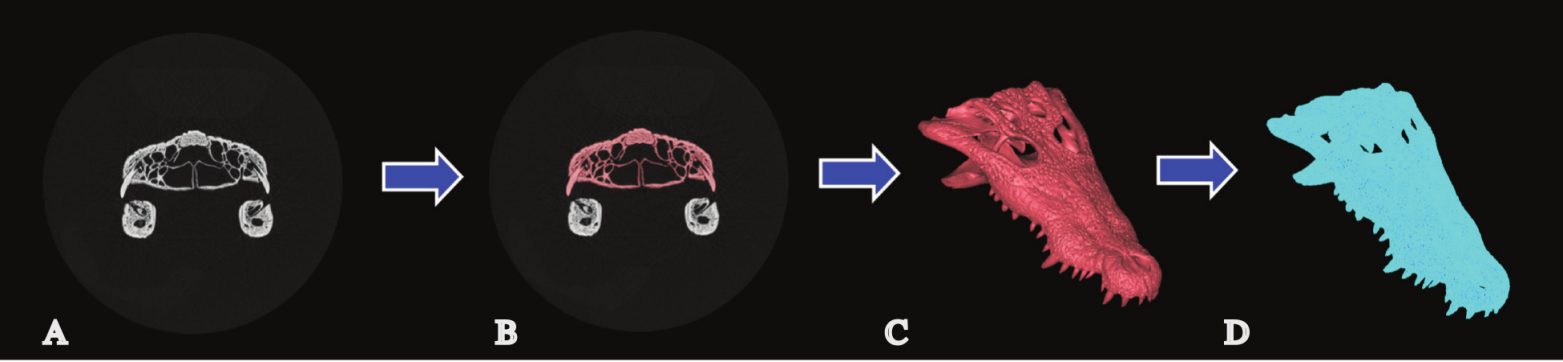
Mesh generation. The process of solid mesh generation from CT data. (A) Single CT slice of a specimen. (B) A masked anatomical feature (the cranium) at a single slice. (C) Generation of a surface model at a given resolution from the image stack. (D) Generation of a solid model from that surface.

Finite element analysis relies on areas of discretised space that divide a complex structure into many simple ones. When the analysis is in 3D, the relevant mesh is a solid (volume mesh). The accuracy of a solid mesh is dependent on the process used to create it. When using CT data this normally involves four steps: (1) The data is acquired through scanning. (2) Anatomical structures of interest are masked (‘segmented’) from the data by selecting relevant pixels from the 2D slices of the scan. (3) A surface mesh is generated from the masked data at a set resolution to generate a surface mesh. (4) This surface mesh is converted into a solid structure composed of a certain number of elements (Panagiotopoulou, 2009; Rayfield, 2007; Richmond et al., 2005) (Fig. 1). The CT data, the surface mesh and the solid mesh must all have sufficient resolution to capture the structure if consistent results are to be obtained (Note that voxel based meshing, where the solid model is generated directly from 2D slice geometry, is an alternate approach to that outlined above.).

Sensitivity analyses track the influence of a specific factor of a model in order to determine its degree of influence on the results. Studies of this type have been undertaken on a wide number of factors used in FEA models including: the way in which the size of models is standardised (Dumont, Grosse & Slater, 2009; Walmsley et al., 2013a), the complexity of the material properties used in the model (Bright & Rayfield, 2011b; Cox et al., 2011; Gröning, Fagan & O’Higgins, 2012; Kupczik et al., 2007; Porro et al., 2011; Reed et al., 2011; Strait et al., 2005; Tseng et al., 2011; Walmsley et al., 2013a), the role of sutures (Bright, 2012; Kupczik et al., 2007; Porro et al., 2011; Reed et al., 2011; Wang et al., 2010), the influence of ligaments (Gröning, Fagan & O’Higgins, 2012; Gröning, Fagan & O’Higgins, 2011), the effect of different orientations of muscle force (Bright & Rayfield, 2011b; Cox et al., 2011; Gröning, Fagan & O’Higgins, 2012; Gröning, Fagan & O’Higgins, 2011; Grosse et al., 2007), the effect of the type of solid elements (Bright & Rayfield, 2011a; Dumont, Piccirillo & Grosse, 2005) and the influence of mesh density (Bright & Rayfield, 2011a; Tseng et al., 2011). These studies have added to a growing collection of information on the effects of model assumptions in FEA. Some of these studies have identified material properties as having a comparatively large influence on the results (Cox et al., 2011; Porro et al., 2011; Reed et al., 2011; Strait et al., 2005; Tseng et al., 2011) where as others have identified that functional aspects of the simulation, such as bite point, are a large influence (Fitton et al., 2012; Walmsley et al., 2013a).

A number of sensitivity studies have previously been undertaken to examine the effects of altering solid resolution on FEA results (Bright & Rayfield, 2011a; Tseng et al., 2011). Using models of a wolf mandible, Tseng et al. (2011) found that 300,000 solid elements were sufficient to produce reaction forces and strain energy close to those of high resolution models i.e., convergence. Higher levels of strain were observed in higher resolution models. Bright & Rayfield (2011a) showed that element sizes of approximately 1 mm edge length (1,250,000 elements) achieved convergence in a pig skull. This study also found that insufficiently dense models underestimated strain and displacement and those areas of high strain converged faster than areas of low strain (Bright & Rayfield, 2011a). Tseng & Flynn (2015) conducted a detailed convergence analyses on a mongoose skull; they found that higher resolution models did not provide more stable outputs, and that convergence patterns differed across constraint types and bite positions simulated. Together these studies show that the number of elements used in comparative analyses does influence results and that the morphological complexity of the structure and loading conditions are important in determining the number of elements required to obtain a convergent result. Consequently the number of solid elements should be sufficiently high in order to be consistent with a convergent result.

The creation of a volume mesh at a certain resolution is only the final step in model generation however. The solid mesh is generated from a surface mesh, and the surface mesh is generated from input geometry (the input geometry is derived from CT scanning, surface laser scanning, photogrammetry, or computer aided design). A high resolution solid mesh requires a high resolution surface mesh to accurately incorporate potential geometric information; any solid mesh resolution exceeding the surface mesh resolution is effectively wasted because it will incorporate a higher number of elements without altering the geometric detail of the structure (although higher resolutions can improve accuracy in the displacement field of the model). Convergence tests on solid mesh resolution are routinely undertaken as part of FEA studies, however, the effect of both input geometry and surface mesh resolution have not been sufficiently investigated. Furthermore, most finite element sensitivity studies have not assessed results in a comparative context. Many studies using finite element analysis include specimens that, because of size or access to data capture equipment, vary considerably in resolution. The effects of resolution at various stages of the finite element model creation process are thus important in a practical sense when planning a study. Here, as a first approach in determining the relative influences of the steps used to prepare a FEA model from computed tomography (CT) data, we aim to examine the degree to which the surface mesh resolution influences FEA results in a comparative dataset of crocodilian crania.

## Materials and Methods

### Data acquisition and surface mesh simplification

**Figure 2:**
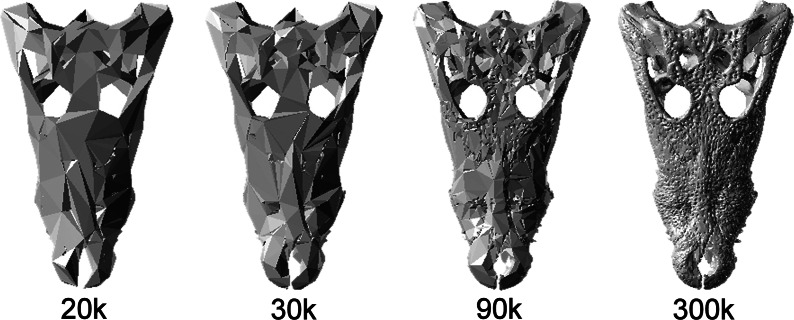
Simplified models. Example of the simplified models. *C. moreletti* models composed of 20k, 30k, 90k and 300k surface elements.

The crania of seven species of crocodilian (comprising *Mecistops cataphractus, Crocodylus johnstoni, Crocodylus intermedius, Crocodylus moreletii, Gavialis gangeticus, Osteolaemus tetraspis* and *Tomistoma schlegelii*) were scanned using computer tomography (CT scanning). These specimens varied greatly in size and morphology, details about the collection of this data can be found in Walmsley et al. (2013b). The software package “MIMICS” version 14 was used to segment the cranium from each scan and generate a high resolution surface mesh of the cranium; the number of elements varied from 600,000 to 3,000,000 depending on specimen size and scanning parameters. Minimal manual editing was undertaken to prevent bias being introduced between models. Using the remesher in the FEA module of MIMICS, the high resolution surface meshes were then down-sampled in number of elements using the “Reduce” function to approximately 20,000 (20k), 30,000 (30k), 90,000 (90k) and 300,000 (300k) surface elements for each of the models (Fig. 2).

### Solid meshing and finite element modelling

The various surface meshes were then exported to HARPOON (www.sharc.co.uk) where they were solid meshed to 1,020,000 (±3.3%) tetrahedral (linear four node type) elements each. Note that solid mesh density was kept constant between models. The c. 1 million element models have much higher equivalent resolutions than most of the surface meshes used to construct them, and were approximately equivalent to the resolution of an 180,000 element surface mesh. In comparative biological FEA normal practice is to use solid meshes that ‘match’ the input geometry/surface mesh resolutions used in the study; however, to do this here would have resulted in solid meshes that differ considerably in solid element size, and this would introduce another variable into our study that is highly likely to confound the effects of different surface mesh resolutions. Bite and Shake load cases were undertaken using the linear static solver in Strand7 (www.strand7.com). Isotropic, homogeneous material properties were used to represent the material properties of crocodile bone (Young’s modulus = 13,471.0 MPa, density = 1,500 kg/m^3^, Poisson’s ratio = 0.3) (Walmsley et al., 2013b). Bite and shake load cases were undertaken on unscaled (natural size) models as well as models that were scaled to the volume of the median model (*M. cataphractus* 90k element model). Rescaling allows for the results to be compared whilst controlling for model size. Identical forces of 30 N were applied to both the natural size and volume scaled models. As both force and volume were standardised this will also conserve force/volume ^2/3^ (Dumont, Grosse & Slater, 2009). The forces applied have been chosen arbitrarily; the use of linear static solves within this study however means that any difference in input force would scale linearly with the resulting strain values.

**Figure 3:**
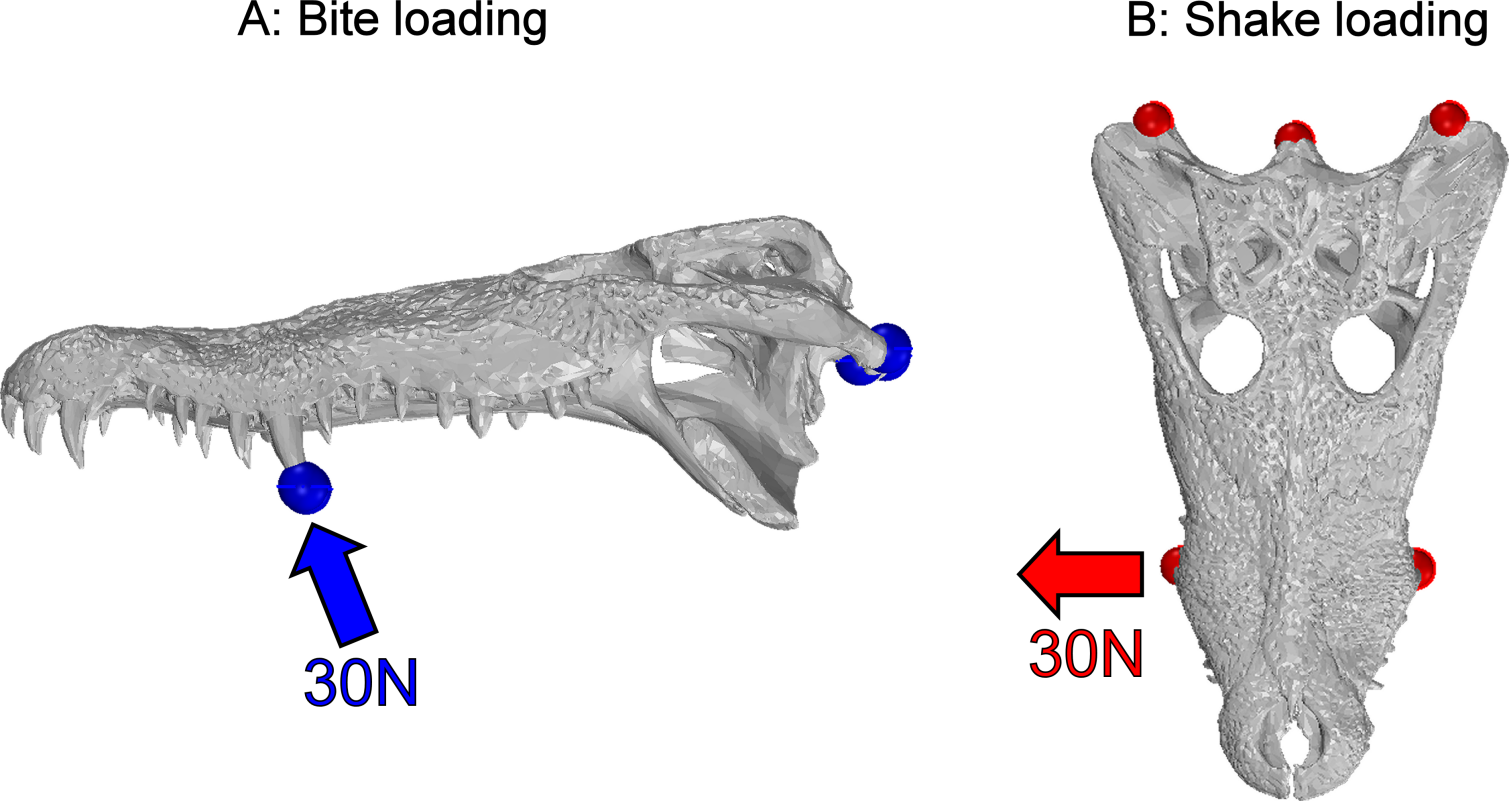
Load cases. The two load cases undertaken within this study. Bite loading where a force of 30 N was applied to the teeth in an arc around the jaw joint (A) and Shake loading where a force of 30 N was applied laterally from the middle teeth (B). Red and blue markers depict the site of restraints; the arrows show the direction in which force is applied.

**In bite loading** a force of 30 N was applied to the largest teeth in the middle section of the tooth row in an arc around the jaw hinge axis. The model was restrained by preventing movement of three nodes in all three axes of translation and rotation; the three nodes were located in the centre of the articulator surfaces of each quadrate and the occipital condyle (Fig. 3A). The location of restraints was standardised between specimens as much as possible, and a patch of stiff beams (Young’s modulus = 200,000 MPa; Poisson’s Ratio = 0.25; density = 7,850 kg/m^3^; diameter = 1 mm) were used at the points of loading and restraint to prevent point loading artefacts; these restraints and beam networks follow model construction techniques used elsewhere (McHenry et al., 2007; Walmsley et al., 2013b; Wroe et al., 2010).

**In shake loading** a force of 30 N was applied to the same teeth in a lateral direction. The model was restrained at the three nodes as described above (Fig. 3B). Stiff beams were used to distribute loads at both the loading and restraint points, as detailed above.

These load cases represent scenarios that, whilst not physiologically realistic, will generate resulting stress and strains of sufficient complexity for use in this study. In reality bite loading would be driven by the jaw muscles, and in extrinsic loading these muscles would also act to brace the skull (McHenry, 2009; McHenry et al., 2007). Loading the models with more realistic forces by including muscle beams would likely significantly influence the patterns of stress and strain observed in the models. Because of this, readers must exercise caution whilst interpreting the results in a biological sense. Differing the type of loading and model constraints (e.g., muscle driven vs. simplified loading undertaken here) is however unlikely to alter the interpretation of the influence of surface geometry on FEA results. Von Mises strain values of each tetrahedral element were exported from Strand7 for each load case. 95% strain values were then calculated by removing the top 5% of strain values using R (R Core Team, 2013). This value represents a way to compare the upper end of strain that is occurring within the models without the inclusion of artefactual data that is often present in maximum strain values (Walmsley et al., 2013b). Walmsley et al. (2013b) shows that the 95% values within many of the same specimens included in this study closely match alternative performance measures such as mean, 75% or 90% values, but not maximum values, which include artefactual peak values, producing a qualitatively different pattern between specimens. Percentage differences were calculated from unscaled models by comparing the 95% strain value of each model to the 95% strain values from highest resolution model of the same species.

## Results

**Figure 4:**
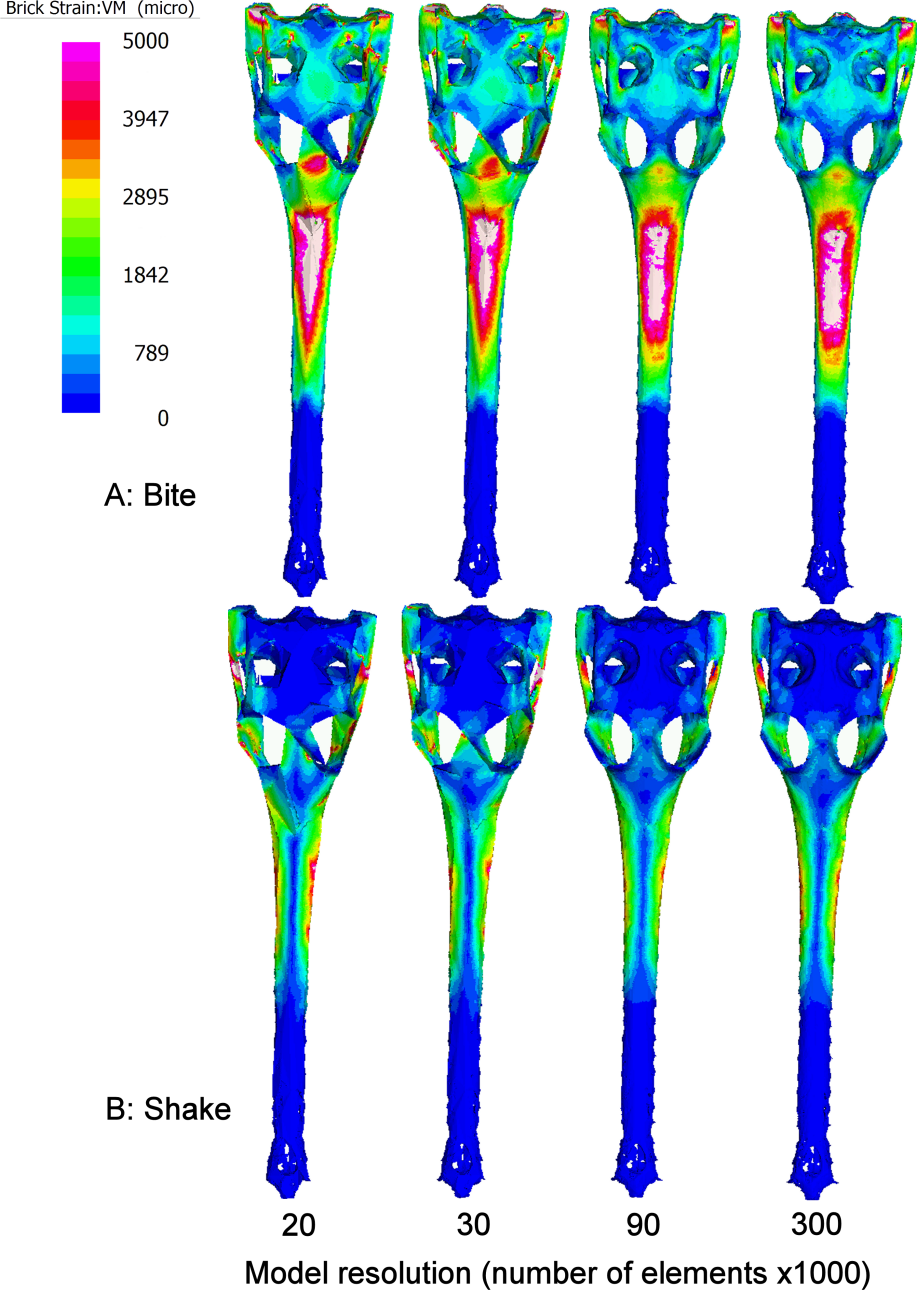
Strain patterns. Variation in VM strain patterns with resolution in *Gavialis gangeticus* during Shake (A) and Bite (B) load cases. Models of 20k, 30k, 90k and 300k surface elements were examined. The colour on the surface of the model represents the level of VM microstrain at that location. Hotter colours represent areas of higher strain than cooler colours. Areas coloured white represent those that exceed 5,000 vm microstrain.

The pattern of strain over the surface of the models varied both in terms of location and magnitude as a result of surface resolution (Fig. 4). Areas that were most affected by mesh resolution included: the skull roof, the area in between the orbits and the area around where the teeth were loaded (Fig. 4).

**Figure 5:**
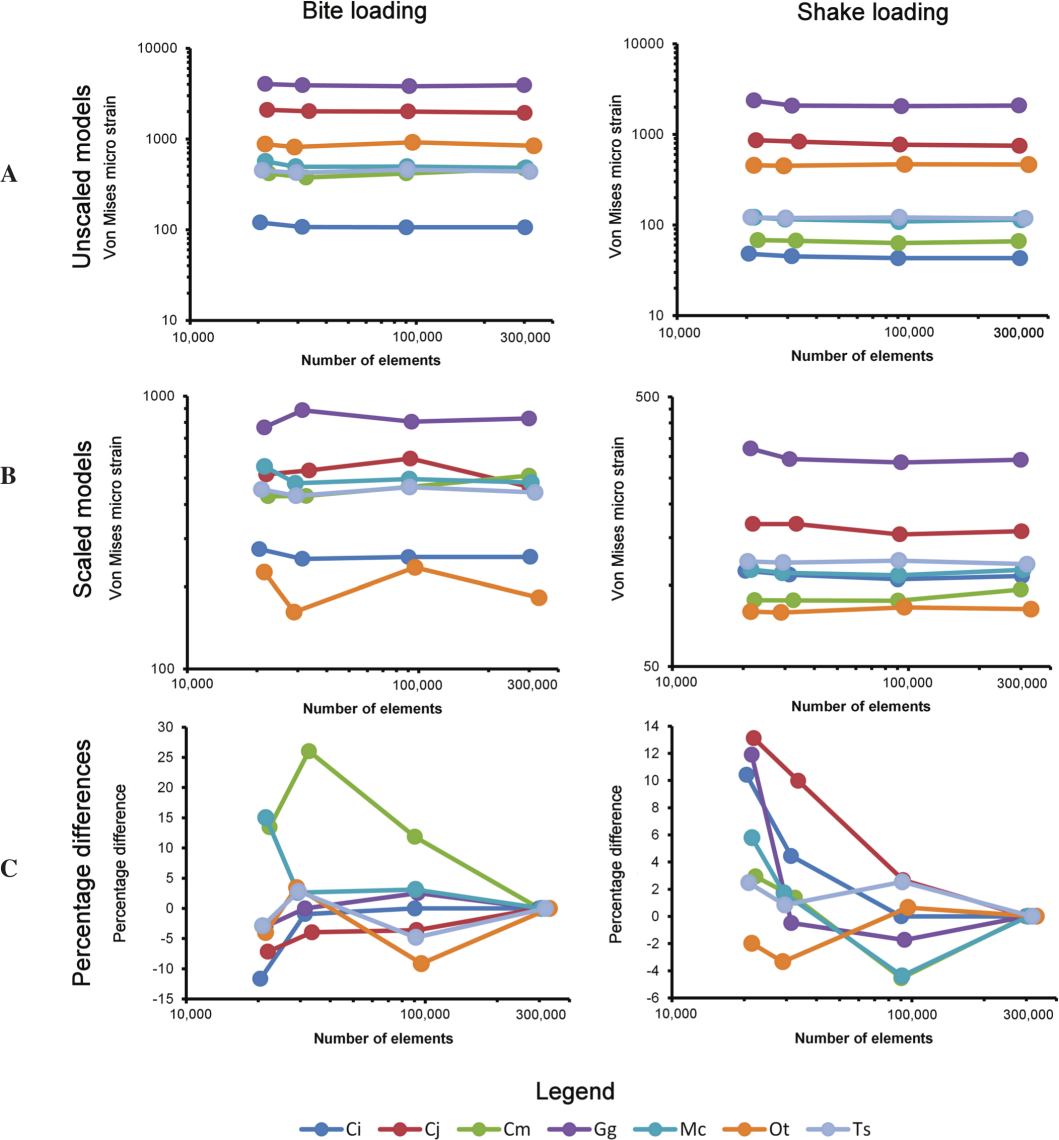
The effect of resolution. (A) The effect of model resolution on 95% vm strain values in unscaled models (natural size). Both *X* and *Y* axes are log10. (B) The effect of model resolution on 95% vm strain values in volume scaled models. Both *X* and *Y* axes are log10. (C) The effect of model resolution on the percentage difference of each of the lower resolution models (90k, 30k and 20k surface element models) to their higher resolution counterpart (300k elements). Only the *X* axis is log10. Taxon abbreviations: *Ci, Crocodylus intermedius; Cj, Crocodylus johnstoni; Cm, Crocodylus moreletii; Gg, Gavialis gangeticus; Mc, Mecistops cataphractus; Ot, Osteolaemus tetraspis*; Ts, *Tomistoma schlegelii.*

Both natural-sized and volume scaled sets of models maintained a similar relative order despite large decreases in surface mesh resolution (Fig. 5). The effects of both size and shape are depicted in the graphs of unscaled models (Fig. 5A). The highest levels of strain in both bite and shake cases was exhibited by the lowest resolution model of *Gavialis gangeticus.* Comparing between all models, Strain values varied between 106 and 4,040 micro-strain (µ*ϵ*) in bite loading and between 43 and 2363 µ*ϵ* in shake loading in natural-sized models. Despite large differences in model resolution the pattern (rank from lowest to highest strain) of results remained very similar (Fig. 5A).

Volume scaling the models produced results showing the effects of mesh resolution on the pattern of results without the influence of size. The process of scaling resulted in the level of strain between specimens becoming more similar (Fig. 5B). This resulted in the pattern of results between models altering slightly with surface resolution. Within the bite load cases four models changed in rank in the 90k, 30k and 20k resolution models. Within the shake load cases all models maintained a constant pattern despite alterations in surface resolution. Strain values varied between 162 and 888 µ*ϵ* in bite loading and between 79 and 321 µ*ϵ* in shake loading (Fig. 5B).

**Figure 6:**
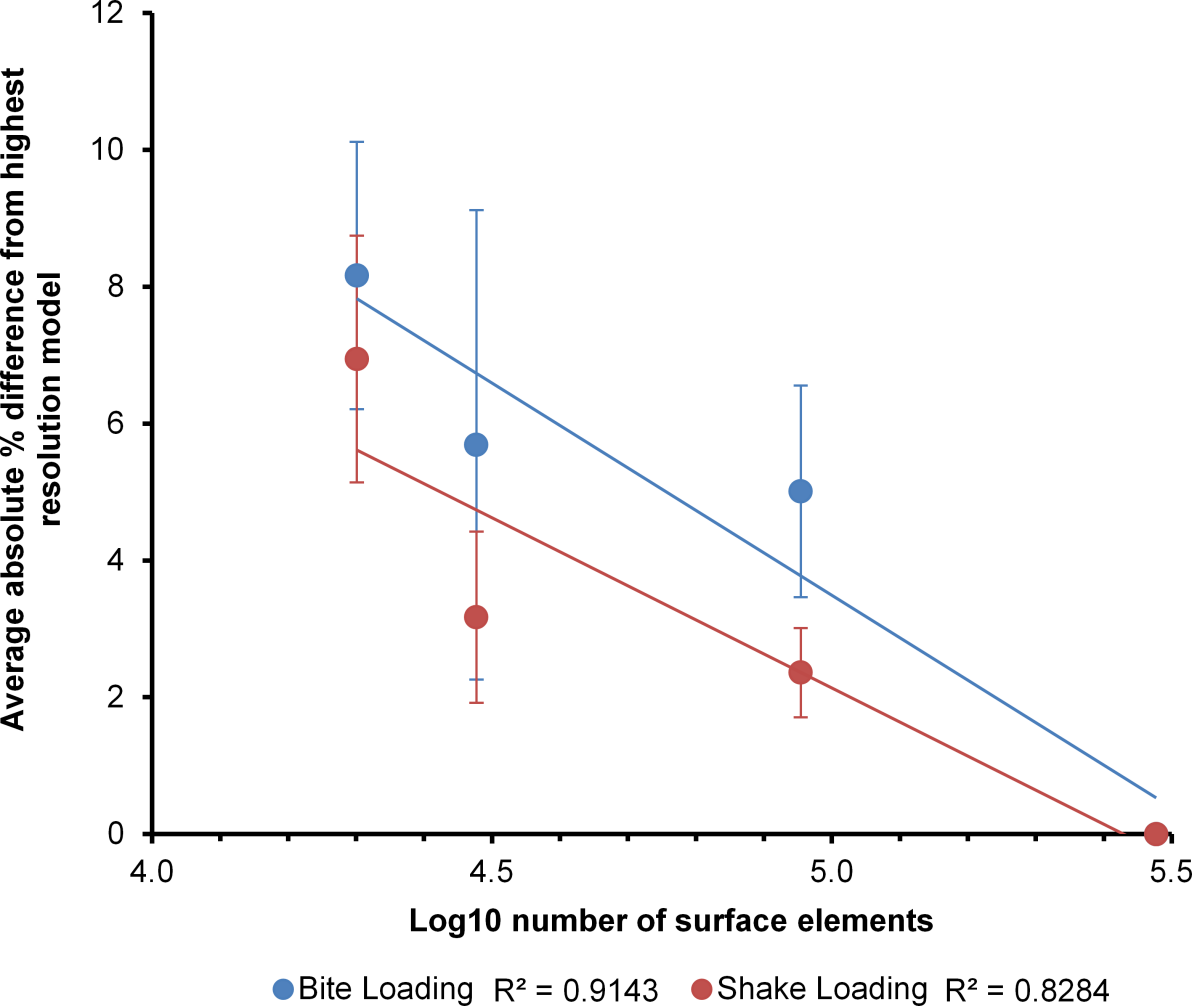
Absolute percentage difference. The relationship between surface mesh resolution and model convergence calculated as average absolute percentage difference from the highest resolution model. Note that the trend lines are only produced from four data points and the results should be viewed with caution.

Comparing the percentage differences in von Mises strain between the highest resolution of each specimen (300k surface elements) and each of the lower resolution models (90k, 30k and 20k surface elements) revealed the effects of surface mesh resolution on the results of each model (Fig. 5C). When simplifying models to a greater degree the model sometimes increased in strain and sometimes decreased. The largest difference between high and low resolution models was 26%, between the highest resolution and 30,000 element *Crocodylus moreletii* model (Fig. 5). Plotting the average percentage difference between the various resolutions revealed a linear relationship between surface resolution and log10 percentage difference in results (Fig. 6). A drop in resolution of approximately 15 fold resulted in only 10 percent difference in results.

## Discussion

Surface resolution was found to have a distinct influence on the results of the finite element analysis. Both the locations of strain and magnitudes of strain varied as a result of resolution; this highlights the need to consider the resolution of the surface data prior to solid meshing for use in finite element analysis. Size and shape were however found to have a larger influence on the results than that of the range of surface resolutions tested (Fig. 5), lending support for the use of a comparative approach in biomechanical analysis.

The location of strain varied as a result of resolution; areas under strain that were of high geometric complexity such as the medial sides of the orbits were the most highly affected (Fig. 4). Bright & Rayfield (2011a) reported that the area that took the longest to converge in a domestic pig skull was close to a region of high geometric complexity. In order to compare FEA results to validation data their study only measured strain at a number of locations where strain gauges could be fitted. The results of this study support the findings of Bright & Rayfield (2011a) and highlight that the level of geometric complexity of a model should be considered when determining whether surface resolution is sufficient.

The high variation in strain observed between the specimens used in this study is likely related to the large amount of interspecific variation present in size and morphology. The *Gavialis gangeticus* specimen which exhibited the highest levels of strain in both biting and shaking load cases is far smaller in size (approximately 190 mm in dorsal cranial length) and has a far more gracile, elongate morphology compared to many of the other specimens used in the study. The simplified loading methods used in this study may also have contributed to the high levels of strain observed. Loading the skulls with forces for simulated muscles, rather than with extrinsically applied loads, may considerably decreased the strain levels observed.

This study employed low order solid elements (Tet4s) to make up the solid mesh. These elements exhibit displacement behaviour described with linear equations and omit constant state stress and strain over each element volume. Whilst these types of elements can perform poorly in some bending applications, the approximately 1 million solid elements used within each of the models here provided sufficient resolution to result in many solid elements comprising each of the major bone structures, resulting in fairly complex strain fields (Fig. 4). Previous studies have shown a high level of convergence in models where this is the case (Bright & Rayfield, 2011a; Dumont, Piccirillo & Grosse, 2005).

When simplifying the model to a greater degree the strain magnitudes sometimes resulted in higher levels of strain and other times resulted in lower levels of strain (Fig. 5). This result is intuitive considering that when simplifying a model the meshing algorithm is forced to choose whether to fill an area with material or not to. Previous studies of mesh resolution have found that strain was most often underestimated in models of insufficient resolution (Bright & Rayfield, 2011a). The findings of this study did not agree with this, with approximately half of the models increasing in strain and half decreasing (Fig. 5). This inconsistency could be a reflection of the use of a single model within previous analyses or a result of methodological differences (e.g., the standardisation of solid mesh resolution within this study or the use of 95% strain values instead of maximum and minimum principal strain as a measure of model performance) between the two studies. Natural sized models were used in this section as several studies have noted that the choice of methods when scaling may influence results (Dumont, Grosse & Slater, 2009; Walmsley et al., 2013a). The location of constraints in the models was chosen with a high level of care; however we must note that the resolution of the models may have altered the morphology of the surfaces at these locations, which may influence the strain patterns observed. There were clear relationships between surface resolution and FEA results. Decreasing surface resolution resulted in an average difference of approximately 8% in VM strain values in the lowest resolution models (approx. 20k surface elements), compared to values from the highest resolution meshes (Fig. 6). This is quite a small effect considering the large decreases in resolution that were undertaken. Ideally our models would have been validated in order to provide a benchmark of the real level of strain in each load case, this is a clear opportunity for future research. Because boundary conditions can also alter strain distributions and magnitudes it is likely that appropriate mesh resolutions may differ in other studies. Furthermore, it is important to note that the crocodilians used within this study have robust bone structure compared to other taxa; caution must be used when trying to extrapolate these results to taxa that have thinner bones and hence may be more susceptible to differences in surface resolution. Future studies should consider resolution at the scanning, surface meshing and solid meshing stages in relation to the specific structures and parameters being assessed.

Models with a surface resolution of 30k elements converged well with those with higher numbers of elements, indicating that the usefulness of higher resolutions would be small. We suggest that the results also have interesting implications for data collection methods in the context of a comparative analysis. Quite simple geometries were found to perform adequately in this instance. Importantly, the implications of this study for collecting data in lower resolutions rely on the assumption that down sampling surface data will result in a similar geometric simplification to initially collecting the same data in low resolution. Although this makes logical sense, future studies will need to confirm this for their taxa, or anatomical features of interest.

The advent of new scanning technologies has resulted in some studies scanning specimens at quite high resolution (Dumont, Piccirillo & Grosse, 2005; Oldfield et al., 2012; Tseng, 2009; Walmsley et al., 2013b; Wroe, Lowry & Anton, 2008) providing solid meshes of over 2.5 million elements. Although this may be necessary in the examination of the biomechanics of fine morphological features, it appears that low resolution data is sufficient for the examination of large morphological differences. FEA studies should consider whether scan, surface and solid mesh resolution is appropriate to answer the questions of the study; our results suggest that very high resolutions are not always necessary for meaningful comparative analysis.

The question of scanning and modelling resolutions is particularly important for comparative studies that may include material that is logistically difficult to scan using high-resolution CT. Although the influence of scan resolution is not directly tested within this study, the results show that, for the specimens and loading scenarios examined here, the level of geometric detail required to obtain reasonable results is quite low and hence low resolution data collection methods could potentially be employed. However, this result is may not hold true for studies interested in finer morphological variation such as most intraspecific comparisons; further analyses will need to be undertaken before we can determine what constitutes a large enough variation in morphology. The measure of performance analysed here (95% VM strain) may also be under less influence by surface mesh resolution than other performance measures (e.g., stress values) or levels of stress or strain in smaller localised areas of the skull.

This result has important implications for data collection and FEA model construction, in that collecting data in lower resolutions would speed up both data collection and solve times. Many fossil specimens, due to their size, fragility or density, are logistically unsuitable for high resolution CT scanning but if lower resolution surface meshes produce meaningful results then the scope for including fossils specimens is greatly increased; photogrammetry, surface scanning, or CAD approaches can be used to generate suitable input geometry, and can be used with specimens where CT data is unavailable or unsuitable. For incomplete or deformed fossils (i.e., the majority of vertebrate fossils), digital reconstruction is required prior to finite element analysis, and these techniques are much easier to use in lower resolution (McHenry, 2009). Our results suggest that low resolution models used in this way will still produce meaningful results. Lower resolution models are also easier and faster to construct, solve, and analyse.

This study adds to a body of literature on the influences of assumptions in FEA models. The results document the effects of altering surface resolution and show that it is possible to get reasonable results for a lower resolution surface if the solid elements are standardised. This result also provides a basis for the use of simplified models in comparative biomechanics. If studies are interested in large morphological differences, such as those tested here, then considerable differences in surface resolution will not influence the “take-home” results of a study.

## Supplemental Information

10.7717/peerj.988/supp-1 Supplemental Information 1 Raw data Raw data showing the number of surface elements and 95% strain values for each of the load cases.
